# Quadrilateral plate fractures

**DOI:** 10.1007/s00402-024-05698-4

**Published:** 2024-12-16

**Authors:** T. Freude, Axel Gänsslen, D. Krappinger, J. Lindahl

**Affiliations:** 1 University Hospital for Orthopaedics and Traumatology, Müllner Hauptstraße 48, Salzburg, A-5020 Austria; 2 Department of Trauma Surgery, Hannover Medical School, Hannover, Germany; 3 Department of Trauma and Orthopedics, Johannes Wesling Hospital, Minden, Germany; 4 Department of Orthopaedics and Traumatology, Medical University Innsbruck, Anichstr. 35, Innsbruck, A-6020 Austria; 5 Department of Orthopaedics and Traumatology, Helsinki University Hospital and University of Helsinki, Helsinki, Finland

**Keywords:** Acetabular fracture, Quadrilateral plate fracture, Anatomy, Classification, Biomechanics, Stabilization concepts

## Abstract

During the last two decades, extended scientific interest focused on quadrilateral plate (QLP) fractures as part of common acetabular fractures. The QLP corresponds to the medial wall of the acetabulum, and different fracture pattern of Letournel´s fracture types are associated with concomitant QLP fractures. Except anterior and posterior wall fractures, all other fracture types may be associated with QLP fractures. QLP fracture features include simple fracture lines up to highly comminuted fractures. A detailed preoperative analysis of these fractures is important to get a better understanding of intraoperative decision making. No consensus exists regarding the optimal classification and treatment of QLP fractures. Various operative approaches and treatment concepts exists depending on the specific QLP fracture type and the acetabular fracture type. Several new implants were development for optimal but often individual stabilization concepts. The gold-standard is still some medial buttressing during internal fixation predominantly using plates, but also screw fixation is considered an option. Additional dome impactions must be considered as an integral part in any QLP fracture analysis and stabilization.

## Introduction

Fractures of the quadrilateral plate (QLP) are usually associated with medial femoral head subluxation and are at risk for additional marginal impaction injuries of the acetabular dome, especially in the elderly patient group. These fractures can have simple fracture pattern or may be highly comminuted [10, 29, 41, 47, 56].

In a first literature review, White et al. analyzed reports dealing with acetabular fractures and central hip dislocation [56]. This search strategy was based on fractures of the quadrilateral plate which in the majority of cases is associated with an acetabular fracture and central femoral head displacement if forces acting lateral on the hip joint. Most often associated fracture types, e.g. associated both-column fractures (ABC), anterior column and posterior hemitransverse fractures (ACPHT), posterior column fractures, transverse fracture types including T-shaped fractures are commonly observed with fractures of the quadrilateral surface [1, 22, 32].

White et al. presented an overview of several treatment options of “medial wall” fractures [56]. Analyzed data for conservative treatment mainly consisted of reports of the pre-Letournel era. Not surprisingly, a high rate of poor results with secondary osteoarthritis were observed. No analysis of fracture types was possible, as the Letournel classification became gold-standard in the final period of these analyses. Analysis of operative procedures predominantly included case descriptions < 10 cases with a large variety of stabilization measures (lag screws, percutaneous screws, plates and screws, cerclage wires, cables). Only four analyses presented between 18 and 35 cases [3, 24, 30, 37].

Chen et al. stated, that in ABC fractures, after fixation of the ilium, “Then, the dome fracture, the posterior fracture and the displaced quadrilateral plate were reduced and fixed with double 1.6 mm cerclage wires under traction of the femoral head, and finally, one or two reconstruction plates and screws were used supplementally to fix the fractures and any comminuted fragments” [3]. Moushine et al. only analyzed percutaneously inserted anterior and posterior column screws without addressing the quadrilateral surface [37]. Laflamme et al. described the technique of infrapectineal plating to address different acetabular fractures with involvement of the quadrilateral plate [30]. Keel et al. introduced the Pararectus approach and only stated, that it is possible using this approach to reduce and fix fracture with quadrilateral surface involvement [24].

All four papers only reported involvement of the quadrilateral plate in acetabular fractures, without giving detailed information on fracture planes, fracture lines or specific quadrilateral treatment options.

In the other cited papers, some osteosynthesis were performed without a detailed description of fracture types and stabilization techniques. Especially, the quadrilateral surface fracture was incompletely described and addressed [56].

The aim of the present analysis is to review the anatomical description, biomechanics, classification and results of treatment in associated quadrilateral plate (QLP) fractures.

## Anatomy and definition of the quadrilateral surface/plate

**Fig. 1:**
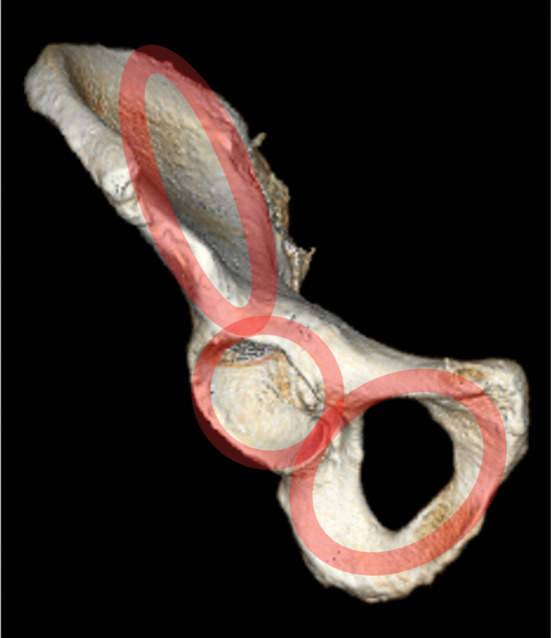
Three-ring structure of the hemipelvis

The acetabular cavity can be considered as a ring construction. Based on the 3-ring structure of the hemipelvis [11], an iliac ring, an acetabular ring and an obturator ring can be identified (Fig. 1).

According to Wolf´s law [57], in the future a real ring structure will be expected.

The acetabular ring consists of the original articular joint, consisting of the facies lunata with the surrounding bone, divided into the anterior wall, the thicker posterior wall, the superior dome and the central acetabular fossa, with a reported thickness of 3–4 mm in female cadavers [7].

**Fig. 2:**
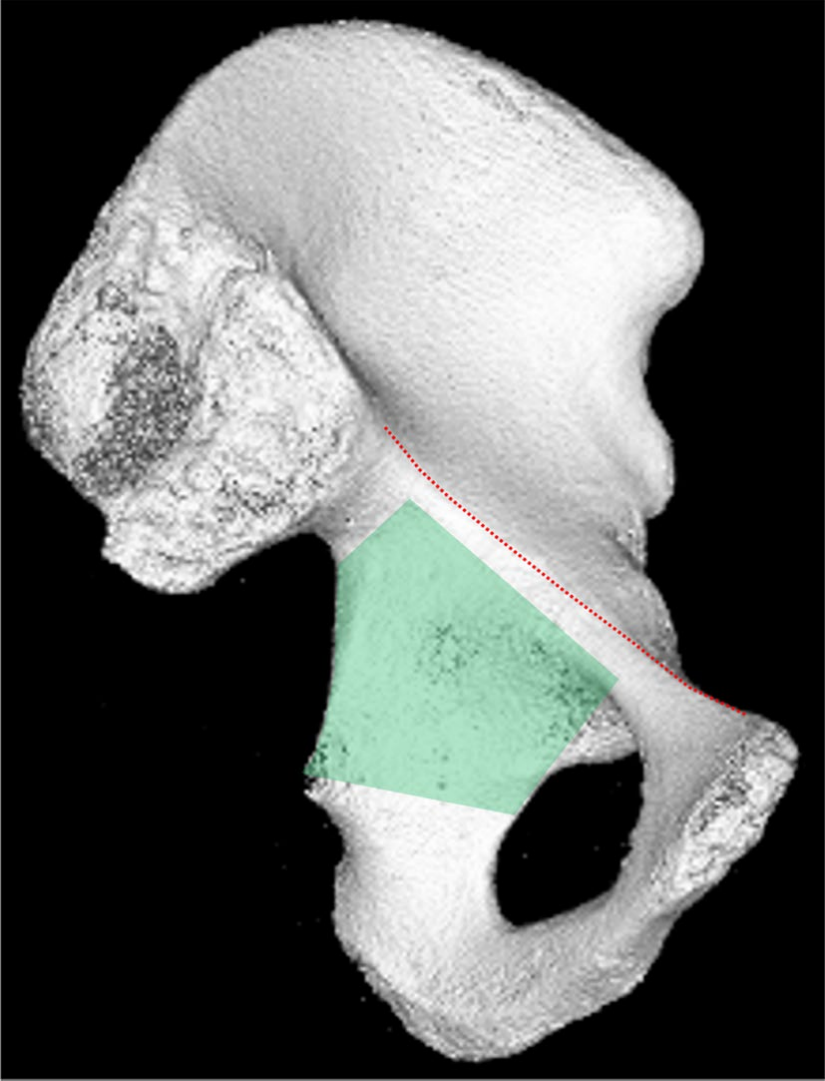
QLP area (green) in relation to the pelvic brim/arcuate line (dotted red line)

The quadrilateral plate refers to the medial wall of the acetabulum with an age dependent thinning [14]. It has a trapezoidal shape with four borders [9, 62] (Fig. 2):

- anterior → line from the posterior border of the obturator foramen to the iliopectineal eminence.
- posterior → zenith of greater sciatic notch along the border to the ischial spine.
- superior → a parallel line just below the pelvic brim up or close to the sciatic buttress.
- inferior → midpoint posterior obturator foramen to the ischial spine.

Centrally, the QLP is extremely thin. In an CT-based analysis of the medial wall of the acetabulum, Stein et al., described the thinnest point of the medial wall to be only 0,6 − 1,2 mm [51]. This was confirmed by Werner et al. who described an average thickness of 1,08 mm but reported a thickening in acetabular dysplasia with pathological values of either the lateral center edge angle (< 20°) or the acetabula index (> 12°) [55].

Guo et al. reported an increase of the reduced cortical thickness area with increasing age [14]. While the quadrilateral surface area remains nearly identical in patients 18–40 years, 41–60 years and above 60 years, respectively. The thin cortical thickness region increased by 3,6% and 24,5%, respectively.

### Radiology

Historically, Köhler´s teardrop figure defined the inferior medial wall of the acetabulum. The teardrop figure is formed laterally by the medial part of the acetabulum (acetabular fossa) and medially by the antero-inferior part of the QLP.

The teardrop figure is not an anatomical structure but a radiological structure, consisting of a very long, almost straight medial line, a large lateral arch and a short-curved line, which connects the caudal parts of the other two lines [26, 38, 42]. Thus, the classical tear drop figure, defined by Letournel, has to be questioned being part of the QLP.

The development of the tear drop figure shows an age dependent course with increasing narrowing [38]. Already in 1977, Kölbel et al. described the relevance of the Judet views to analyze the QLP with shape differences of the tear drop Fig. [27]. In their anatomical study, the tear drop figure corresponds more to the antero-superior edge of the QLP.

**The classical teardrop figure on the radiological AP view does NOT fully correspond to the QLP.**

In 2018, ElNahal et al. radiologically (conventional views, CT) defined the anatomy of the QLP using a titanium mesh based on the described anatomical landmarks [9].

**Fig. 3:**
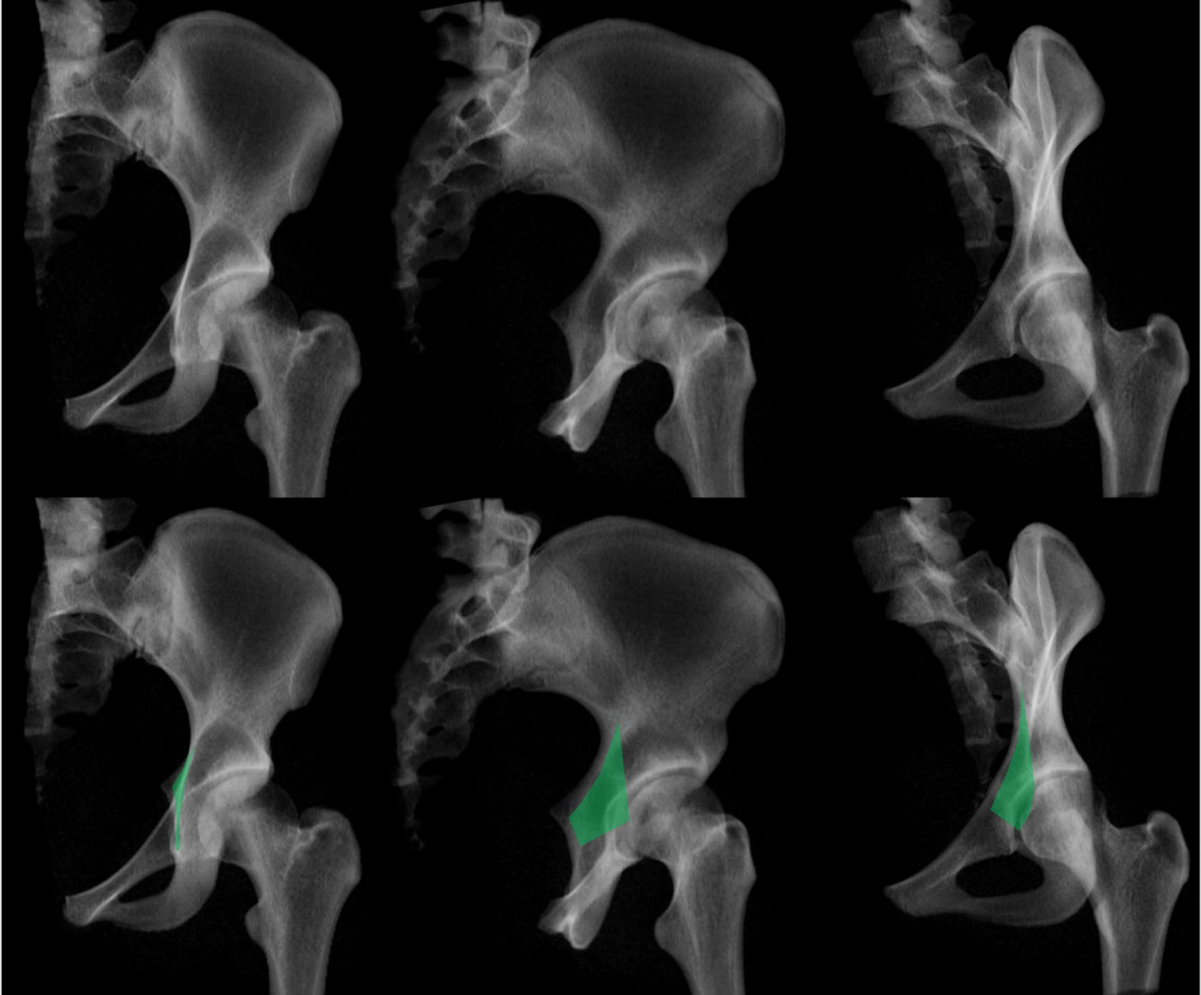
True radiographic appearance of the. QLP on standard acetabular views (AP view, iliac oblique and obturator oblique view)

On standard radiographs, AP view, iliac oblique and obturator oblique views, the QLP was defined as (Fig. 3):

- AP view: small longitudinal area between the iliopectineal an ilioischial line extending from the level of the AIIS level to the distal end of the tear drop figure.
- Iliac oblique view: a warped trapezoid is seen with a broader base and an acute angle superiorly.
- Obturator oblique view: a comparable warped trapezoid shape projects on the anterior column.

On axial CT´s, the QLP corresponds to the whole medial wall.

Understanding the anatomy of the QLP implies where to place and fix implants. Guy et al. already identified a posterior safe zone inside the true pelvis for screw application to avoid intraarticular screw penetration. Depending on the size of the femoral head, four to six plate holes have to be left free in the periarticular region [15].

**Fig. 4:**
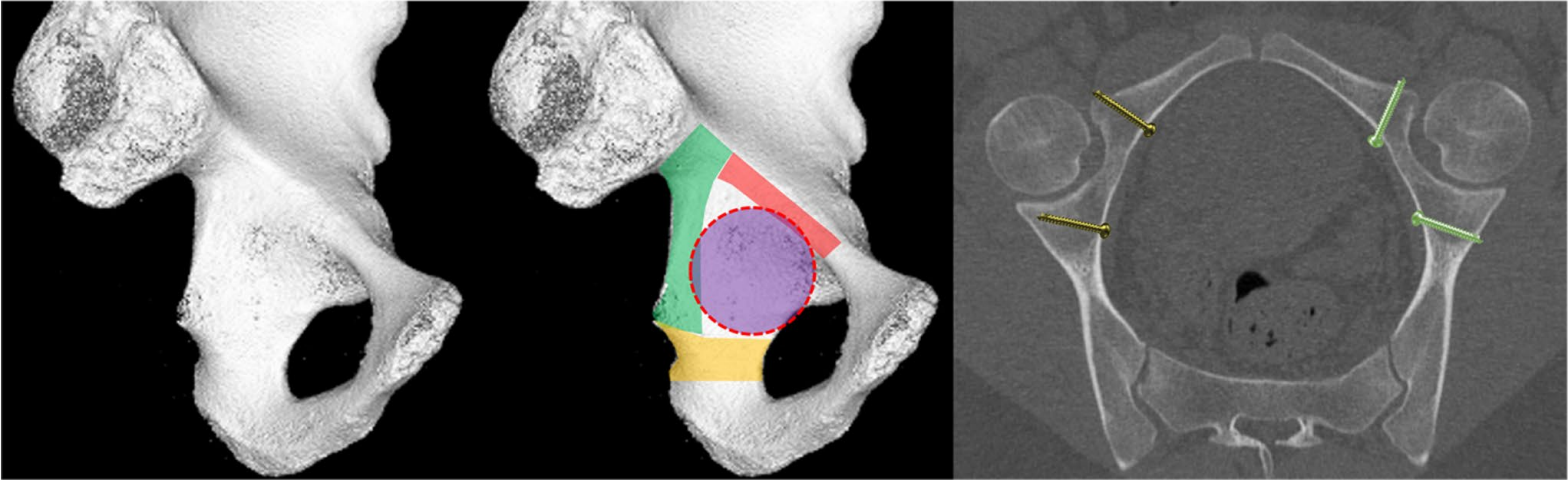
Safe zones for periacetabular screw application: posterior column (green), inferior (yellow), and close to the arcuate line (red). Acetabulum cavity (purple). Screw orientation perpendicular to QLP (golden screws) and away from the joint (green screws)

In an anatomic study in Chinese people, He et al. analyzed an extended safe zone around the quadrilateral surface using different stabilization methods [16]. Parallel 1 cm cuts in relation to the pelvic brim were performed. Posterior-superior (1 cm below the pelvic brim) an average safe distance of 30 mm was found, and below the zenith of the sciatic notch down to the ischial spine a safe distance of 12,4 mm was reported. These distances match with the results of Guy et al. [15]. An additional horizontal zone below the inferior acetabular level was reported, but no data on the width were reported [16]. Possible screw orientations were drawn into a model (Fig. 4) Optimal and safe screw application in this safe zone is perpendicular or away from the risk zone. This was confirmed in presented clinical data.

## Classifications

In Letournel´s classification, the basic analysis consists of fracture detection of the anterior and posterior column and/or wall or fractures with a transverse fracture component [21, 31, 32]. Injuries to the QLP are not part of this classification. Several authors recently published classifications of quadrilateral plate fractures. This led Zhang et al. to propose a “3-column” classification for acetabular fractures consisting of an anterior and posterior column and integrating a roof column [64]. The definition of these columns is according to the classification of acetabular fractures proposed by Rowe and Lowell in 1961 [44], where the acetabular region was anatomically divided into three areas, which corresponded to the primary ossification centers. Thus, an anterior pubic segment (inner wall), a posterior ischial segment (posterior acetabulum), and an iliac segment (superior dome) were distinguished.

Within this classification, QLP fractures are predominantly observed in associated both and three column fractures [54].

In a first attempt to analyze quadrilateral fracture pattern, a 3D-CT evaluation was performed in 84 patients with acetabular fractures. A 3D medial, slightly oblique view was generated for analysis and definition of the quadrilateral involvement. Two fracture entities were described [40]:

- T-fractures: the fracture starts at the superior acetabular dome or at the pubo-acetabular junction of the anterior column; these fractures were divided into single anterior column involvement (incomplete transverse fractures), involvement of both columns from the medial view (transverse fracture components) up to more complex T- or Y-shaped fractures with a secondary „vertical“ fracture line running towards the obturator foramen.
- C-fractures: the main fracture line is a high anterior column fracture line extending below the level of the pelvic brim to the pubic area; the fracture severity increases from a minimally displaced posterior hemitransverse fracture line showing a triangular quadrilateral fracture area, to a more displaced fracture up to pelvic brim comminution and displaced posterior column component.

In 2018, ElNahal et al. classified QLP fractures into three groups and an additional theoretical fracture type based on CT-findings [9, 62]:

- Type QLP 1: complete separation from the anterior column, partial separation from the posterior column, both with simple fracture lines.
- Type QLP 2: type 1 with anterior comminution.
- Type QLP 3: comminuted QLP fracture with separation from the anterior and posterior column.
- Type QLP T: simple trapezoid fracture completely separated from both columns.

**Fig. 5:**
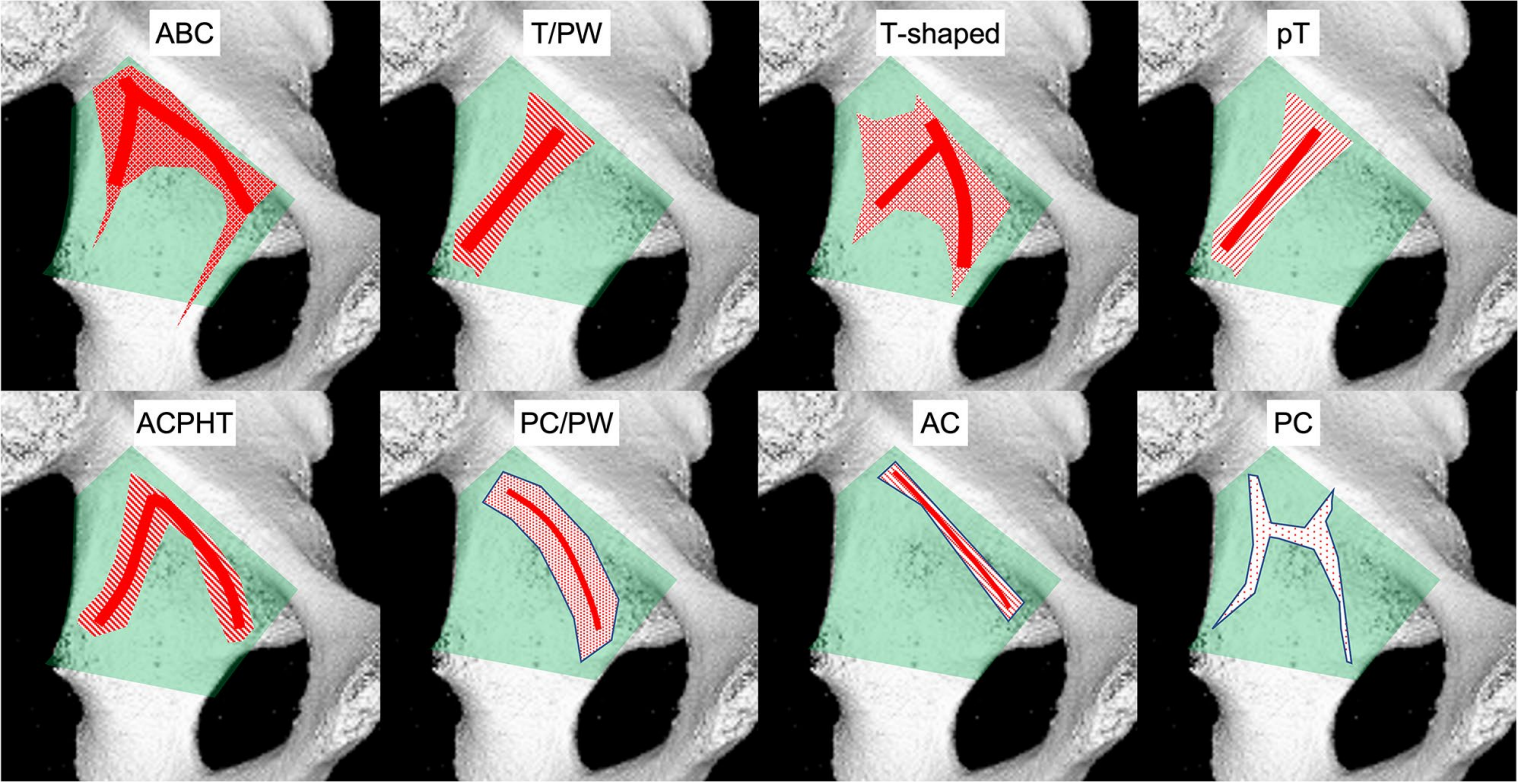
Fracture mapping results in 8 of 10 specific acetabular fracture types according to Letournel, with drawing of the main fracture orientations

In 2018 and 2019, Yang et al. performed a fracture mapping analysis [61] and correlated fracture types according to Letournel with specific quadrilateral surface involvement (Fig. 5) [62].

Based on these results upper transverse QLP fracture lines (parallel to the pelvic brim), perpendicular fracture lines (to upper transverse fracture lines), upper and lower oblique and anterior vertical fracture lines were identified.

Chen et al. recently proposed another classification system consisting of four fracture types. The basis of this classification is involvement of the classical acetabular columns (A = anterior column, P = posterior column) and the amount of QLP fracture (C = QLP involvement, partial (1) or complete (2)) [5] resulting in six different possible fracture types. As also suggested by El Nahal et al., a fourth fracture type was added isolated QLP fracture (partial or complete). The definitions of the different fracture types are as follows:

- AQ = fractured anterior column and intact posterior column with complete fracture line parallel to the pelvic brim and partial/incomplete fracture line perpendicular to the first line from the posterior column [17].
- PQ = fractured posterior column and intact anterior column.
- APQ = fractured anterior *and* posterior column; the QLP is always completely separated from the anterior column and partial (APQ-1) or complete (APQ-2) from the posterior column.
- Q = isolated QLP fragments with intact columns.

This classification showed high agreement between individual and different observers [5].

Isolated QLP fractures are extremely rare. Only 6 cases are reported in the literature since 1987 [8, 13, 29, 33, 36, 60] (Table 1).

**Table 1:** Case summary of isolated QLP fractures

Author	Year	Age	Sex	Mechanism	Fracture	Treatment
Meinhard	1987	27	m	motorcycle	central acetabular fracture defect	ORIF femoral neck
Laflamme	2009	31	m	MVA	central fracture, FH impaction	fragment excision
Douraiswami	2012	22	f	stair fall	undisplaced central fracture	conservative
Makwana	2018	74	M	seizure	bilateral	ORIF: suprapectineal plate + 90° reco-plate
Xiao	2018	28	f	fall from toilet	medial FH fracture dislocation	3D customized plate
Gulija	2023	25	f	seizure	incomplete L-shaped fracture, dome impaction	suprapectineal Stryker plate

## Recognition of the quadrilateral plate fracture

Medial buttressing to prevent medial femoral head subluxation was advised. Medial buttressing was first described as an adjunct by Qureshi et al. [41]. A combined suprapectineal and infrapectineal plating construct was performed for centrally displaced acetabular fractures, thus additionally addressing the quadrilateral plate fracture.

Sen et al. in 2013 first focused on multifragmentary quadrilateral plate fractures and stated, that „inadequate reduction and stabilization of quadrilateral plate fractures leads to incongruous joints and early arthritis“ [47]. A 90°–90° plate construct was favored with a classical suprapectineal plate and an additional 90° bended T-plate underneath the suprapectineal plate placed above the iliopectineal eminence to buttress the quadrilateral surface. The chosen approach was an iliofemoral approach with ASIS osteotomy and an additional medial window (3rd window of the ilioinguinal approach) [47]. Using this concept, in 36 patients, 83,3% anatomic and 16,7% near anatomic reconstructions could be achieved, resulting in 77,8% good and excellent clinical and radiological at latest follow-up. In two patients (5,5%) secondary THA was necessary [47].

**Fig. 6:**
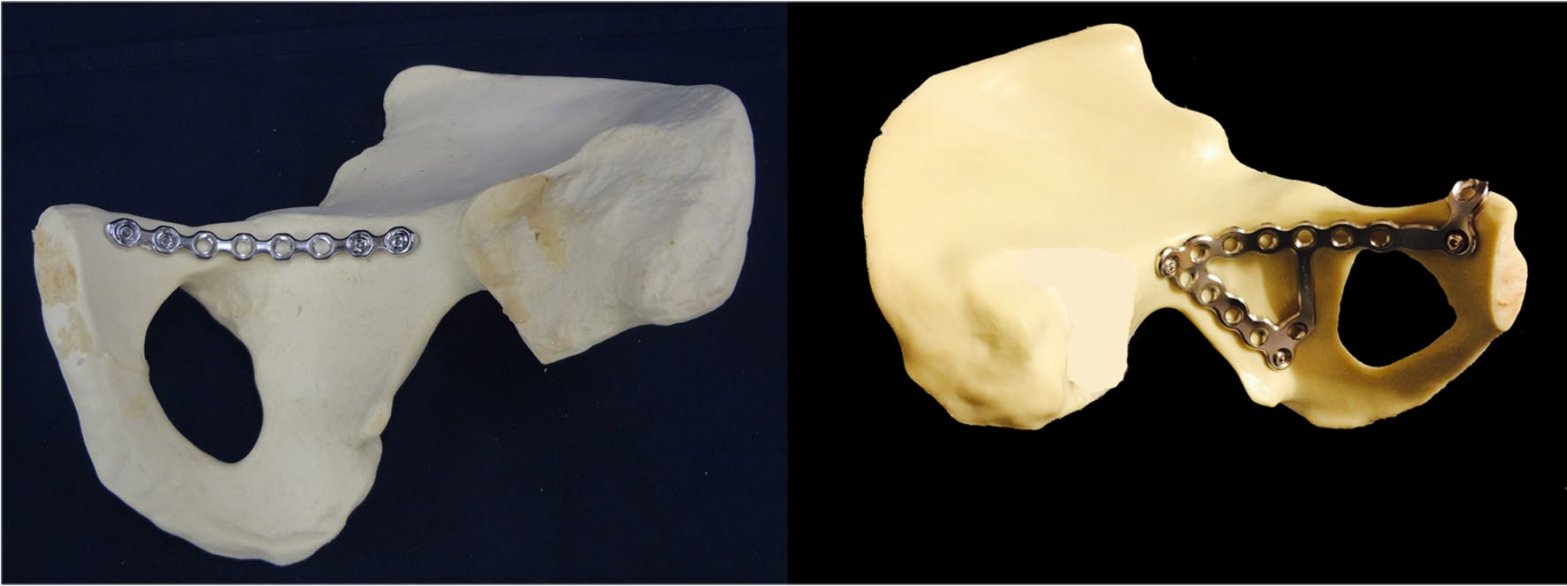
Classical infrapectineal buttress plating concept with a simple reconstruction plate or using a newly designed plate

**Early reports already recommended medial buttress plating of acetabular fractures with concomitant QLP fractures** (Fig. 6).

Exemplary, Rommens et al. described the treatment results in 40 patients with anterior fracture types (ABC, ACPHT, AC) and T-shaped fractures [43]. Primary THA was performed in 7,5% due to associated fracture modifiers indication worse prognosis. After a median time interval of 36 weeks in additional five patients THA was necessary due to non-anatomic reduction, secondary QLP displacement, or posttraumatic osteoarthritis. Overall, in 77,5% an anatomic joint reconstruction was achieved.

The main problem of all reports dealing with QLP fractures consists of analyses of different acetabular fracture types, different QLP involvement and therefore result analyses not allowing fracture-type and treatment specific results. The overall reported clinical and radiological results are comparable to well-known summarized fracture analyses.

Thus, for future evaluations, clear concept based analyses should be performed, with defined parameter evaluation and whenever possible fracture-type specific analysis.

## Approach

QLP-fractures are usually fracture parts of specific acetabular fractures. Predominantly, anterior column fractures, ACPHT fractures associated BC fractures and T-shaped fractures are associated with QLP fracture involvement. A wide variety of these fracture types is reported.

Thus, anterior approaches are typically used for addressing these additional fractures, e.g. ilioinguinal approach [34], intrapelvic approach [18, 19], Pararectus approach [24, 34], and iliofemoral approach with/without osteotomy of the ASIS [47]. In some cases even the direct anterior approach (DAA) is an option.

Anterior-based approaches have the advantage of directly addressing concomitant dome impaction through a cortical window or through the fracture line [2, 20, 28, 46].

## QLP reduction

The classical displacement is a medially displaced femoral head creating the QLP fracture with or without additional marginal impaction located at the anterior and superior dome area of the acetabulum.

A sequential reduction concept is favored:

- lateral femoral head traction using a Schanz screw inserted into the femoral neck allowing better mobilization of the QLP fragment(s) and visualization of dome impactions.
- cleaning and mobilization of the QLP fragment(s).
- addressing the dome impaction with reduction and temporary or already definitive fixation.
- direct reduction of the QLP fragment(s) from medial using a ball spike pusher or comparable instruments (intrapelvic approach), asymmetric reduction forceps (ilioinguinal approach).

### Stabilization concepts

Newer reports describe results of treatment in acetabular fractures and periprosthetic fractures with involvement of the QLP [43].

Beside standard reconstruction plates used as infrapectineal (medial) buttress plates [30, 41, 52], newly designed supra- and infrapectineal plates with additional stabilization options to fix the posterior column and support the QLP are used for supporting the QLP [25, 48, 63].

The addition to address the QLP include mesh-plate constructions [14, 45], integrated 90° angulated plate additions [18, 39], and box-plate configurations allowing anterior and posterior fixation relative to the QLP [53]. It has to be considered, that the 90° additional plate options primarily focus on stabilization of the posterior column.

The concept of RE Peter to buttress the quadrilateral plate or the posterior column without fixing the plate within the true pelvis results in inadequate stability, which was confirmed already in a biomechanical analysis [6].

**The present gold-standard is still a plate system fixing the anterior column fracture and fixing the QLP fracture with different intrapelvic plate modifications.**

Screw stabilization has shown adequate biomechanical stability. In a CT analysis the safety of screw application parallel to the quadrilateral surface was analyzed in 100 Chines adults [65]. Three points were defined using the middle window of the ilioinguinal approach based on visualization and palpation of the iliopectineal eminence. From the iliopectineal eminence a perpendicular line was calculated to the arcuate line (pecten ossis pubis). 5 mm medial to the arcuate line a point was defined on this line. Perpendicular from this point the most critical insertion point was defined 13 mm posterior and two other points 1 cm anterior (A) and posterior (B) were calculated from this insertion point. The mean minimum medial, anterior and posterior inclination angles were 6–7°, 0° and 46–47° at point A, respectively. At point B, these angles were 3,5° lateral inclination, approx. 60°, and 3–4°, respectively.

Wu et al., introduced the DAPSQ concept (Dynamic Anterior Plate-Screw system for Quadrilateral plate) for treating concomitant QLP fractures [58, 59] (Fig. 6). This concept was based on the three screw concept of Karim et al., who avoid QLP fragment tilt or rotation with three extraosseous screws placed medial to the QLP fragment(s) [23].

In a first analysis of 32 patients with 19 ABC fractures, 9 ACPHT fractures and 4 T-shaped fractures, anatomic reduction could be achieved in 59,3% and imperfect reductions in 28,1%. After an average of 47 months excellent and good clinical results were reported in 87,5%. No joint penetration was observed [59]. As a logical consequence, a suprapectioneal anatomical plate was developed with an additional plate whole for application of the medial half screw, which showed adequate surgical results [58].

Recently, Wu et al. analyzed 37 patients with different acetabular fractures, predominantly anterior and transverse fracture types [58]. According to the ElNahal classification 18, 13, 6 were classified as QLP1, QLP2 and QLP3 fractures. They distinguished long-term results based on this classification an showed a decrease of good and excellent clinical and radiological results with increasing QLP involvement and severity. Thus, it was shown, that this QLP fracture classification has some prognostic value. It has to be considered, that the relevance of the fracture type could not be analyzed in detail, thus the prognostic value has to be proven in larger studies.

**Fig. 7:**
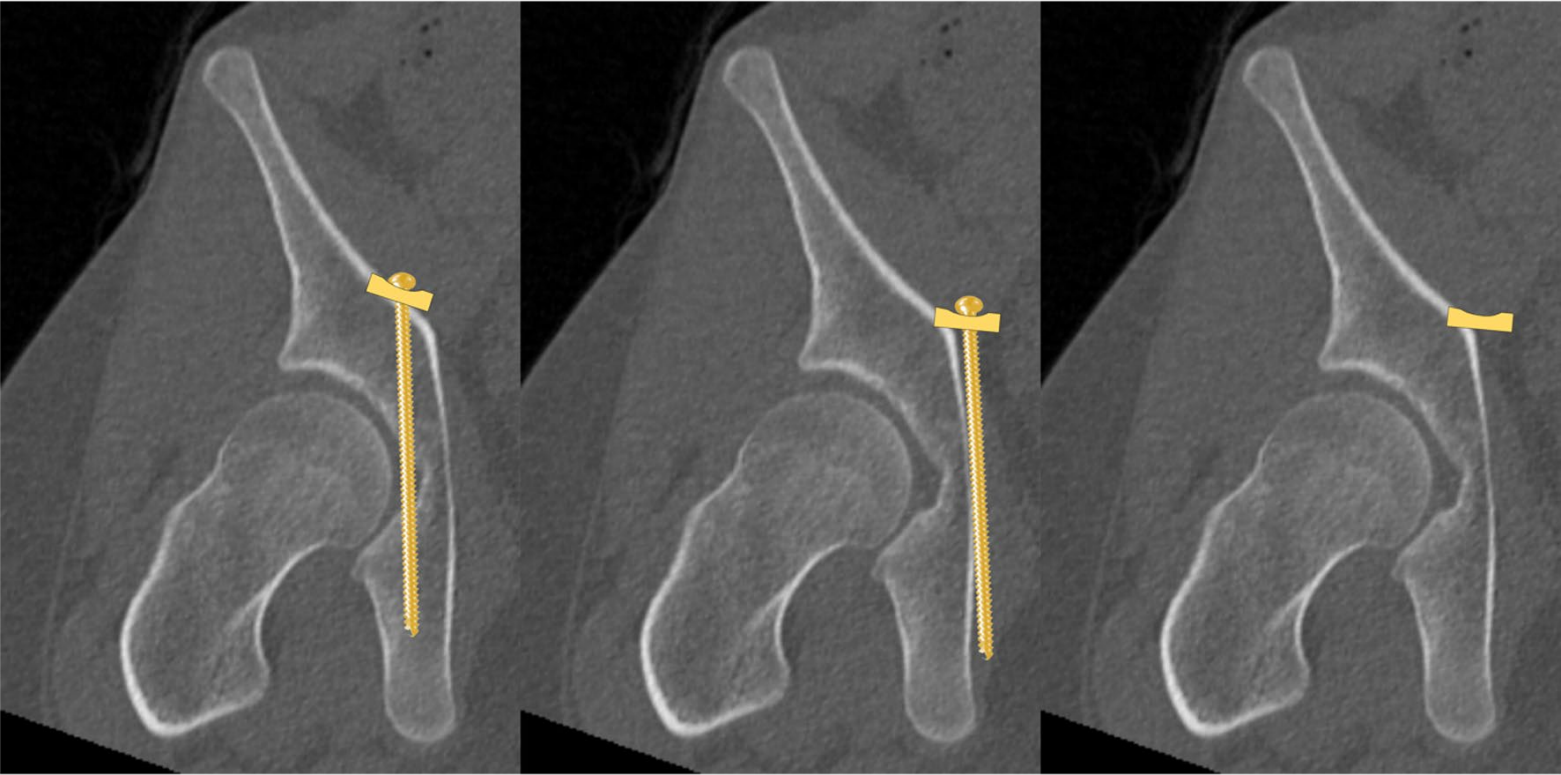
Risk of intraarticular screw penetration and standard plate position (left); concept of extraoseous screw application to avoid medialization of the QLP (middle) and partial osseous fixation according to the Dynamic Anterior Plate-Screw system

The type of treatment of the QLP fracture was performed using a Dynamic Anterior Plate-Screw system for Quadrilateral plate (DAPSQ). This concept includes screw application through a reconstruction plate far medial at the pelvic brim parallel to the medial wall with only 1/3 to 1/2 transverse screw diameter entering the bone to avoid articular penetration (Fig. 7).

**The DAPSQ concept was designed based on the traditional suprapectineal reconstruction concept for acetabular fractures involving the anterior column.**

## Biomechanics

In 2010, Culemann et al. performed a biomechanical study on synthetic and cadaveric pelves after creating and anterior column posterior hemitransverse fracture (ACPHT) in a single leg stance model [6]. Fixation was performed using a curved reconstruction plate with additional periarticular long screws including an infraacetabular screw fixing additionally the posterior hemitransverse component, a reconstruction plate with an underlying H-plate without an infraacetabular screw and without fixation of the H-plate along the quadrilateral plate, a locking plate with shorter periarticular screws and a mash-type locking plate. The latter three constructions did not fix the posterior fracture component. The most stable construct (e.g. less displacement) was the standard suprapectineal reconstruction with additional periarticular long screws, adequately fixing the anterior against the posterior column.

Spitler et al. used 15 synthetic pelvic models after creation of a high ACPHT fracture and simulated a standing position [50]. First an iliac crest lag screw and a supraacetabular lag screw was inserted, followed by suprapectineal plating with each two screws close to the SI-joint and the pubic tubercle. Then one posterior column lag screw was inserted compressing the posterior hemitransverse fracture component. Beside this standard fixation, the set-up conducted of an additional quadrilateral surface plate, 4 long peri-articular screws parallel to the quadrilateral surface into the ischium, and an under-contoured infrapectineal buttress plate, respectively. The construct with long periarticular screws was associated with the highest stiffness. Maximal fracture displacement was observed for the posterior hemitransverse fracture component and the free quadrilateral surface fragment.

May et al. also used comparably to Spitler et al. plastic pelves simulating ACPHT fractures [35]. The stiffest constructs included suprapectineal plate stabilization with periarticular screws along the QLP with and without additional infrapectineal transverse plate fixation.

Chen et al. analyzed ACPHT fractures with an additional isolated quadrilateral surface fragment (QLS) fragment in a simulated reverse standing position [4]. Four fixation constructs were compered: infrapectineal QLS buttress plate, suprapectineal QLS buttress plate, suprapectineal reconstruction plate with 3 periarticular long screws, and infrapectineal reconstruction plate with 3 periarticular long screws. The greatest construct stiffness was observed with the suprapectineal plate + periarticular long screws including an infraacetabular screw. Comparable but slightly inferior results were observed with the infrapectineal buttress plate.

Graul et al. compared the standard suprapectineal plate with an anatomically pre-shaped suprapectineal plate with integration of inner pelvic posterior column plate part [12]. Both plates were tested in a single leg stance model using synthetic bones with and without additional infraacetabular screw fixation. The new 3D plate was superior to the standard plate, while adding an infraacetabular screw to the standard plate resulted in comparable construct stiffness up to the level of 3D plate alone. Adding an infraacetabular screw to the 3D-plate was without relevant increase of stability.

Şimşek et al. in a lateral compression simulation using plastic hemipelvis models could show that the combined supra- and infrapectineal plate fixation with additional IAS provides the most stable fixation of the ACPHT acetabular fractures [49].

**The optimal stability in biomechanical analyses was gained with periacetabular screw fixation of the QLP.**

## Discussion

**Fig. 8:**
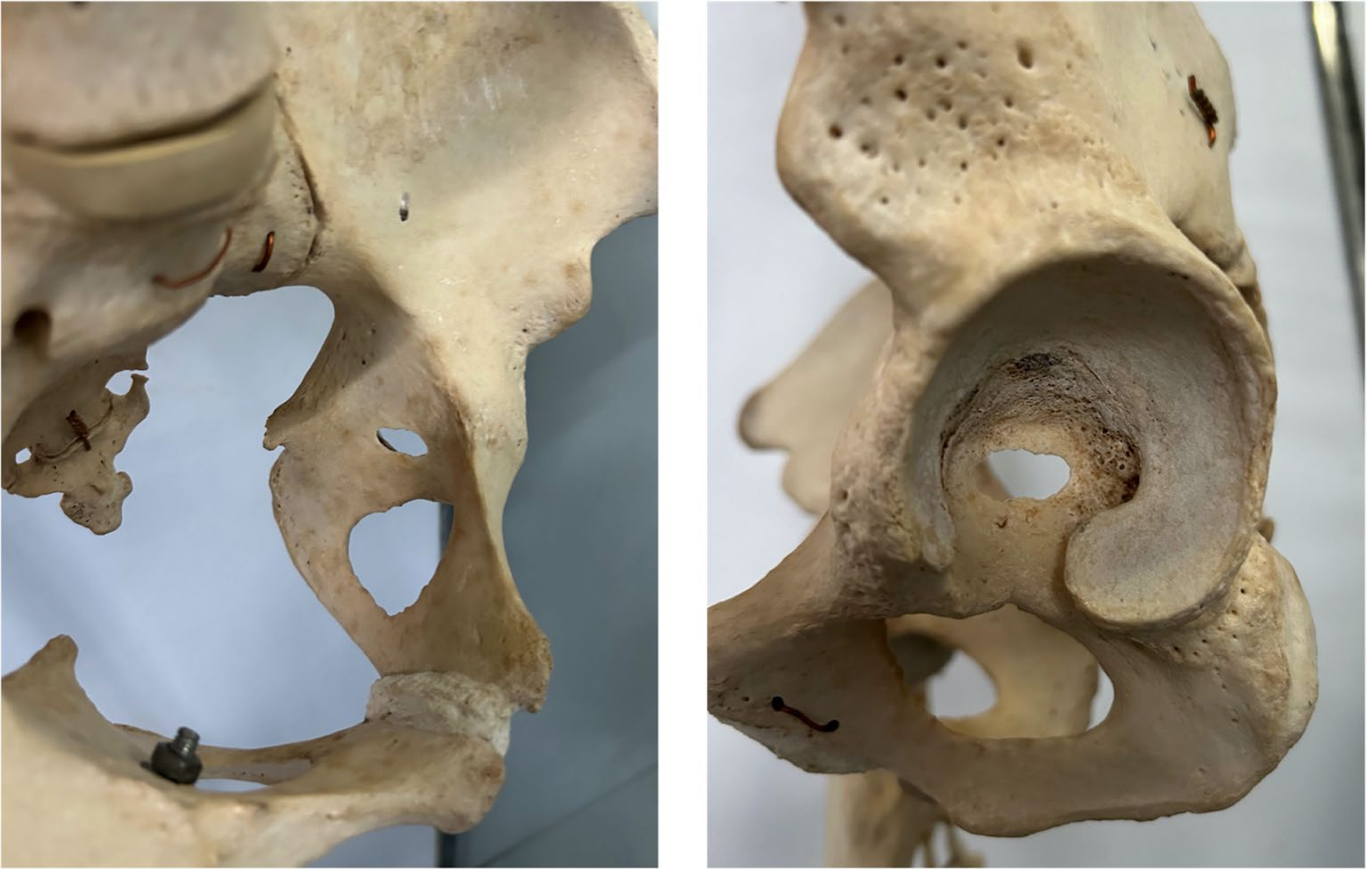
Asian pelvis already showing a central medial wall defect (acetabular fossa) resulting in a ring structure of the acetabulum

The quadrilateral plate is without significance in terms of function, stability and biomechanics of the intact hip joint. From developmental and embryonic history, it is formed by fusion of the three pelvic bones, i.e. the pubic bone, the ischium and the iliac bone. According to Wolff´s law, bone develops and increases its strength resulting in bone density, if stress is acting on the bone. However, if no or only minimal stress acts on a bone, bone will be reduced (Lit). As a logical consequence, it can be assumed, that due to the thinness of the central QLP (Guo 2019), this area has no biomechanical relevance. Several arguments support this fact:

- there are no natural loads during any hip motion acting onto the acetabular floor.
- press-fit anchorage of the cup during hip replacement does not require medial stability, i.e. even if the cup is placed too deep/medial, it will be stable; the acetabulum can act as a wash bowl without a socket.
- in Asian regions, often already a central wall defect is observed (Fig. 8).

Considering these facts, the biomechanical relevance and the anatomical exact extent of the QLP should be questioned in terms of their relevance during acetabular fracture osteosynthesis and especially which part of the quadrilateral surface is clinically relevant.

When treating acetabular fractures with concomitant QLP fracture, two important points may influence the outcome.

**Fig. 9:**
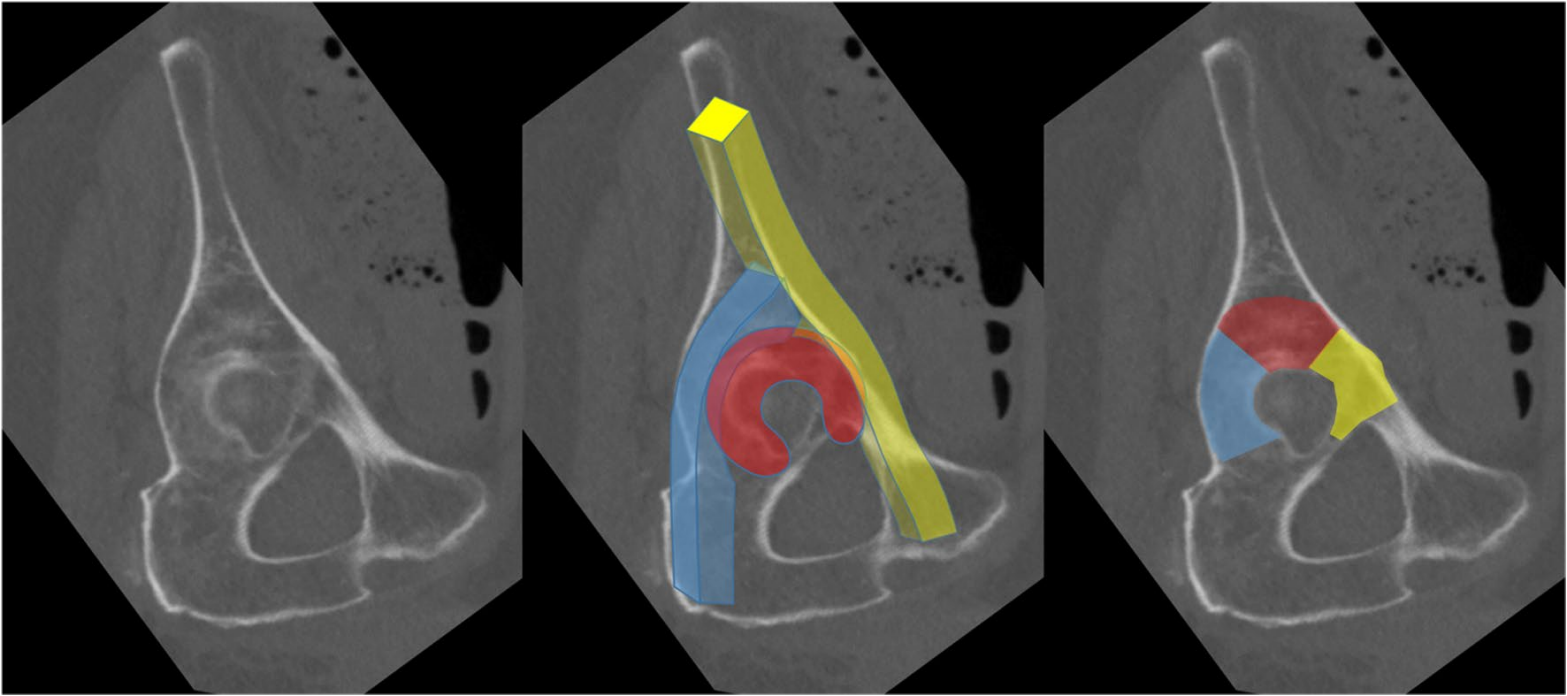
Keystone concept (right): the classical both column bony configuration of the acetabulum (left) with integration of Letournel´s column drawing (middle). The superior dome as a keystone (red) between the anterior (yellow) and posterior wall (blue)

**Fig. 10:**
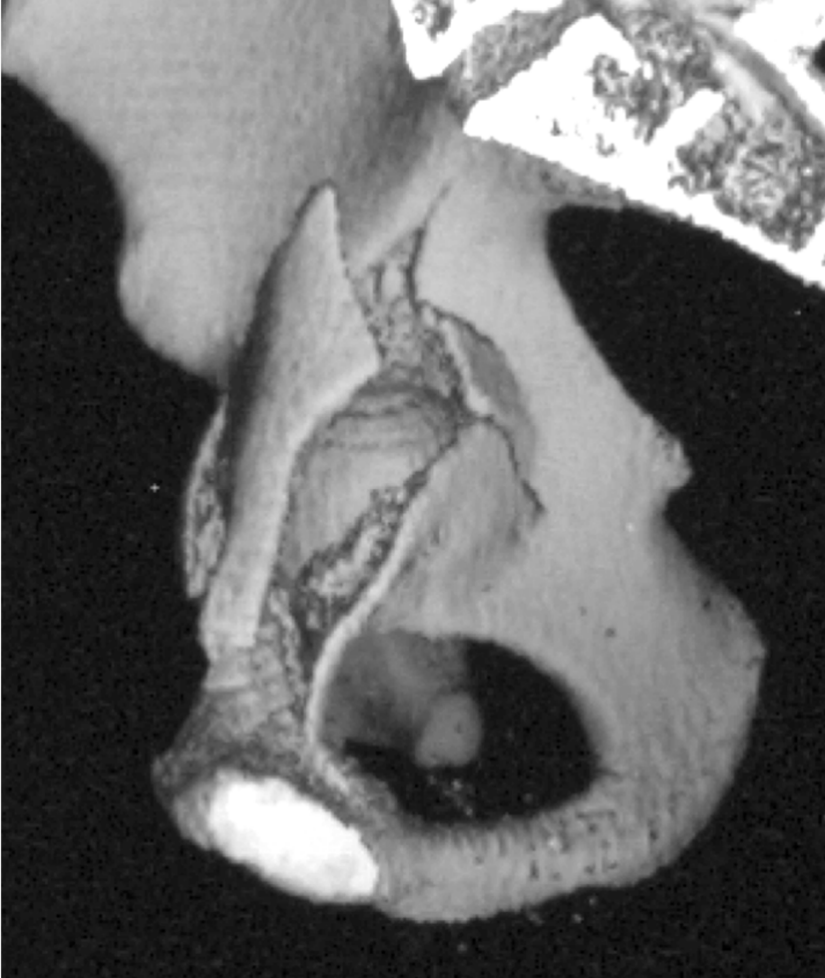
Typical comminuted QLP-fracture with pelvic brim fracture fragment and cranial and medial displacement and intactness of the caudal part of the bone, the latter attached to the posterior column

1. Anatomically, the QLP can be divided into a cranial third and two distal thirds. The cranial third corresponds more to the pelvic brim, than to the QLP and represents a 3rd acetabular column. Comminuted fractures in this area are often associated with superior marginal impaction; the latter can be interpreted a s a key stone between the anterior and posterior column or wall (Fig. 9). This keystone is stabilized medially by the cranial third of the QLP or pelvic brim. In clinical practice, after proper reduction of the dome fragment, the keystone can only be fixed stable, if the cranial 1/3 of the QLP gives adequate support, while the caudal 2/3 of the QLP are irrelevant without contribution to overall stability. In associated comminuted fractures in the anterior wall region together with superior dome impaction, e.g. comminuted fractures of the cranial third of the quadrilateral plate, adequate fixation is mandatory. This is supported by the fact, that these parts of the QLP are often uninjured (Fig. 10).

2. The caudal two thirds of the QLP are of minor relevance and do not provide relevant biomechanical stability, and therefore are without influence the outcome. They are usually connected to the posterior column (Fig. 10), particularly in the case of ACPHT fractures. The posterior hemitransverse fracture component shows usually internal rotational displacement leading to an impingement between the femoral head and the fragment like a pincer impingement with the risk of development of early osteoarthritis, if not adequately reduced.

Intraoperative derotation of the caudal posterior column part can be performed by the attached QLP with medial pressure application during anterior approaches, especially during the intrapelvic approach. Thus, the inferior 2/3 act primarily as a reduction tool. To avoid re-displacement, adequate fixation is necessary. Fixation can be performed using an anatomical wing plate, a column screw or the infraacetabular screw in order to avoid re-internal rotation of the posterior column part.

The concept of the 3-column classification was introduced by Zhang et al. [64].

Instead of using the classical anterior and posterior column concept of Letournel, the former three bones, creating the hemipelvis, present each one column. Thus, the acetabular dome area represents the 3rd column, which was formerly part of the posterior column (Fig. 9).

The roof or dome column is located between the anterior (pubic branch) and posterior column (ischial branch) (Fig. 2) [64].

In detail, the superior dome is located between the anterior (junction between the roof and anterior column) and posterior wall (junction between the roof and posterior column) like a keystone, while the junction between the anterior and posterior column is defined as the medial wall. Zhang et al. defined the acetabular roof as a critical structure for maintaining hip joint stability in acetabular fractures and called it the roof wall. Thus, reconstruction of these three walls is of major clinical importance. The connection of these walls results in a partial ring structure (horseshoe = acetabular articular part).

The medial wall consist predominantly of major parts of the QLP (Fig. 10). According to pelvic anatomy, this medial wall is extremely thin, in average 1 mm or even a hole is existing ([14], and Fig. 8). Thus, its biomechanical relevance must be highly questioned.

This concept fits into the 3-ring structure concept of Gänsslen et al. who supposed an ilium ring, an acetabular ring and the obturator ring [11]. The obturator ring is already a real ring, while the acetabular ring has sometimes already a ring structure and the ilium ring often has an extreme thin central bone part (Fig. 1).

## Conclusion

Until recently, regarding quadrilateral plate fractures, there are more open questions, than answered questions. The quadrilateral plate is now anatomically well defined, but this anatomic structure is not depicted by the classical teardrop figure. Thus, detailed radiological analysis of conventional x-rays and especially CT-scans is mandatory to define the fracture configuration.

Several comparable classifications of QLP fractures were published mainly focusing on attachment of quadrilateral fragments to the anterior and posterior column.

No consensus exists regarding the optimal treatment of QLP fractures. Various operative approaches and treatment concepts exists which led to development of new implants and stabilization concepts. The gold-standard is still some medial buttressing during internal fixation predominantly using plates, but also screw fixation is considered an option.

Additional dome impactions must be considered as an integral part in any QLP fracture analysis and stabilization.
